# Intravascular large B-cell lymphoma masked by multinodular goiter of the thyroid: a case report and diagnostic pitfall

**DOI:** 10.3389/fonc.2026.1850324

**Published:** 2026-07-16

**Authors:** Guona Zheng, Lei Xu, Lili Peng, Tingting Liu, Lin Kang, Huanfen Zhao

**Affiliations:** 1 Department of Pathology, Hebei General Hospital, Shijiazhuang, Hebei, China; 2 Department of Gland Surgery, Hebei General Hospital, Shijiazhuang, Hebei, China; 3 Department of Oncology, Hebei General Hospital, Shijiazhuang, Hebei, China; 4 Department of Heart Center, Hebei General Hospital, Shijiazhuang, Hebei, China

**Keywords:** diagnostic, intravascular large B-cell lymphoma, multinodular goiter, pitfalls, thyroid

## Abstract

Intravascular large B-cell lymphoma (IVLBCL) is a subtype of diffuse large B-cell lymphoma (DLBCL), which is characterized by the proliferation of neoplastic B−lymphoid cells within the lumens of small and medium-sized blood vessels, especially capillaries. It is a rare and highly aggressive malignancy with frequent multi-organ involvement, most commonly affecting the central nervous system, bone marrow, spleen, skin and adrenal glands. Nevertheless, IVLBCL arising in the thyroid gland is extremely rare. Nodular goiter is pathologically defined as thyroid nodular hyperplasia, which is a common benign thyroid lesion. Herein, we report a patient with IVLBCL complicated by bilateral nodular goiter. Notably, the IVLBCL was confined solely to the nodular goiter tissue, with no invasion of the adjacent normal thyroid parenchyma or cervical lymph nodes. Both peripheral blood circulating tumor cell detection and EBER staining were negative. Under low microscopic magnification, a typical background of nodular goiter was observed, and tumor cells were scattered in the intervals of thyroid follicles, mimicking inflammatory lesions. The lesions were almost completely obscured by the background of nodular goiter. This unique and occult growth pattern is highly prone to missed diagnosis, especially for junior pathologists. IVLBCL carries an overall poor prognosis. Early definite diagnosis and timely initiation of standardized chemotherapy can significantly prolong patient survival.

## Introduction

Intravascular large B-cell lymphoma (IVLBCL) is a rare and highly aggressive extranodal subtype of diffuse large B-cell lymphoma (DLBCL), with an incidence of less than 0.5% (1, 2). It was first described by Pfleger and Tappeiner in 1959 (3). The typical pathological features are characterized by massive accumulation of neoplastic cells with B-cell immunophenotype within small vessels, especially capillaries, accompanied by marked endothelial hyperplasia, vascular lumen occlusion, and progressive multi-organ involvement (4). To date, IVLBCL involving the thyroid gland has only been reported in sporadic case reports. The diagnosis of IVLBCL is challenging, as it lacks the classic manifestations of conventional lymphoma, such as lymphadenopathy, space-occupying lesions in peripheral organs, or circulating lymphoma cells. Herein, we present a rare case of IVLBCL confined to thyroid nodules. The tumor cells were restricted to small blood vessels and grew among thyroid follicles, which greatly increases the risk of pathological missed diagnosis. Through literature review and retrospective analysis of the clinicopathological features of this case, we aim to raise pathologists’ awareness of this rare disease and avoid missed diagnoses.

Circulating tumor cells (CTCs) are intact malignant cells originating from solid tumors that shed from primary or metastatic lesions into peripheral blood after detaching from tumor parenchyma and surviving transiently in the systemic bloodstream. CTCs harbor invasive and metastatic capacities and act as critical biomarkers for monitoring tumor progression.

## Case presentation

**Figure 1:**
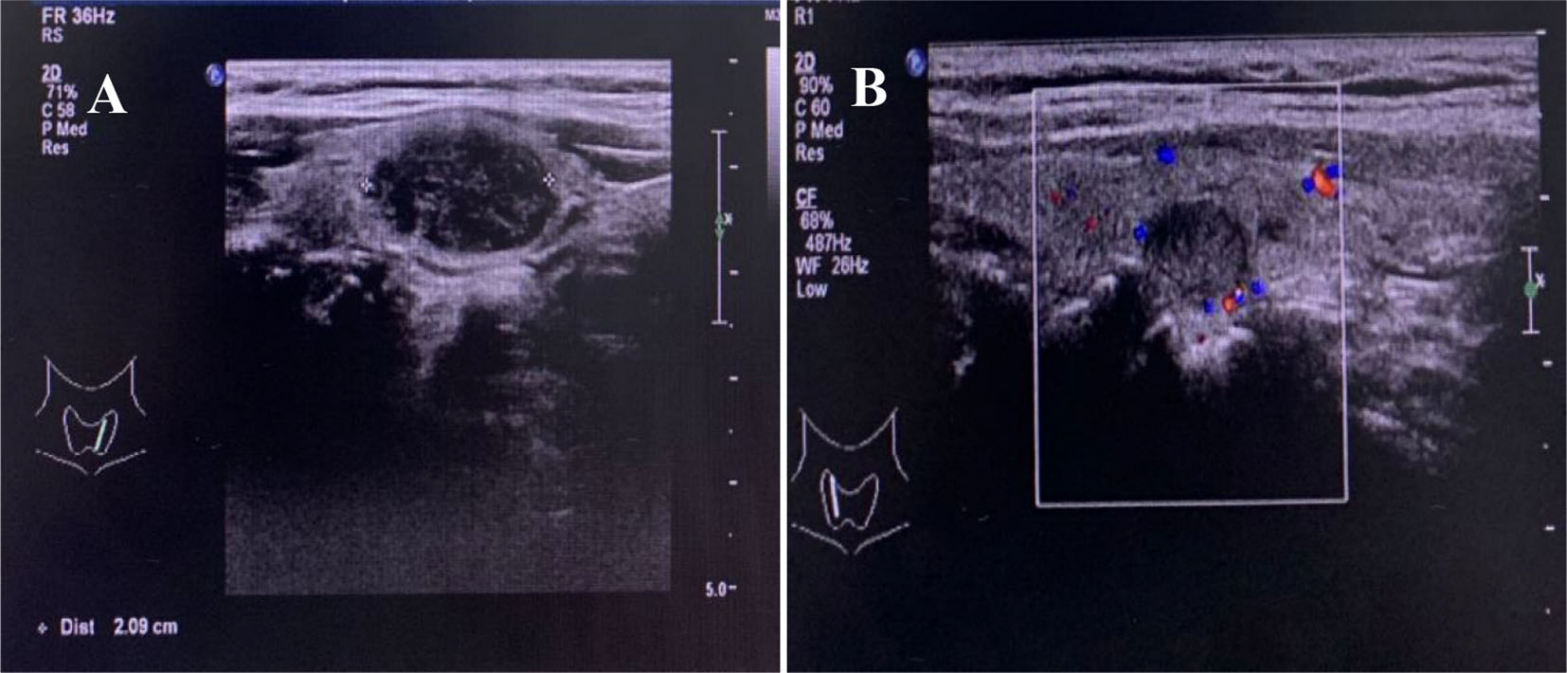
**(A)** A nodule measuring approximately 2.09 cm × 1.66 cm is visible on the left side, suspicious for malignancy. **(B)** A nodule with a diameter of approximately 0.8 cm is seen in the right thyroid lobe, suspicious for malignancy.

A 63-year-old female patient was admitted to the hospital due to bilateral goiter detected on physical examination. Thyroid ultrasonography revealed multiple nodules in both thyroid lobes with diameters ranging from 0.8 to 3.0 cm, and imaging findings were consistent with changes of nodular goiter. A calcified mass measuring 2.09 cm × 1.66 cm was identified in the middle of the left lobe, suggesting a suspected malignant lesion, classified as TI-RADS 4b (**Figure 1A**). A 0.8 cm nodule with malignant signs was observed in the middle of the right lobe, classified as TI-RADS 4a (**Figure 1B**). The patient was hospitalized for thyroid space-occupying lesions. She presented with night sweats but no fever. There were no discomforts such as hoarseness or dysphagia, and no family history of thyroid diseases. No positive neurological signs were noted on physical examination. Admission laboratory results were as follows: Red blood cell count: 3.95×10¹²/L (4.3–5.8×10¹²/L); Hemoglobin: 125 g/L (130–175 g/L); Alanine transaminase: 30 U/L (0–40 U/L); Serum lactate dehydrogenase: 220 U/L (15–240 U/L); Hydroxybutyrate dehydrogenase: 245.0 U/L (15–240 U/L); Total bilirubin: 27.4 μmol/L (5.1–20 μmol/L) and direct bilirubin: 10.6 μmol/L (0.1–10 μmol/L), both mildly elevated. Relative percentages of lymphocyte subsets showed that CD19^+^ B lymphocytes were 19.6% (5.0%–18.0%), and CD3^+^ T lymphocytes were 63.9% (50.0%–84.0%). All numbers in parentheses mentioned above indicate the reference range. Thyroid and parathyroid function tests, including triiodothyronine (T3), thyroxine (T4), free triiodothyronine (FT3), free thyroxine (FT4), thyroid-stimulating hormone (TSH), thyroglobulin (Tg), anti-thyroglobulin antibody (Anti-TG), anti-thyroid peroxidase antibody (Anti-TPO), parathyroid hormone (PTH), and calcitonin (CT), were all within the normal reference ranges. After complete preoperative evaluation, the patient underwent bilateral thyroidectomy combined with bilateral central lymph node dissection.

Isolation and identification of CTCs using the YZY-CTC-A10 instrument. Peripheral venous blood was collected in EDTA-anticoagulated vacutainers and stored at room temperature. All specimens were processed within 2 hours post-collection to avoid cellular degradation. For CTC separation, whole blood was pre-fixed with paraformaldehyde (PFA) to stabilize cellular morphology and dimension. This treatment prevents cell shrinkage or swelling-induced filter pore occlusion and trans-membrane cell leakage, ensuring precise size-based enrichment. The pre-fixed blood suspension was pumped through the filter membrane at a constant flow rate under negative pressure: plasma and red blood cells passed through the filter, whereas CTCs were trapped on the membrane surface. The membrane was further washed sequentially with PBS under negative pressure to eliminate non-specifically bound blood cells and plasma impurities for further purification. Captured cells on the membrane were fixed in methanol prior to membrane retrieval. For subsequent identification, fixed samples received morphological staining, and CTCs were identified via light microscopy.

## Pathological results

This specimen was initially tentatively diagnosed as simple nodular goiter by a junior pathologist. The neglected intravascular atypical lymphoid elements were subsequently discovered following re-examination by a senior pathologist.

**Figure 2:**
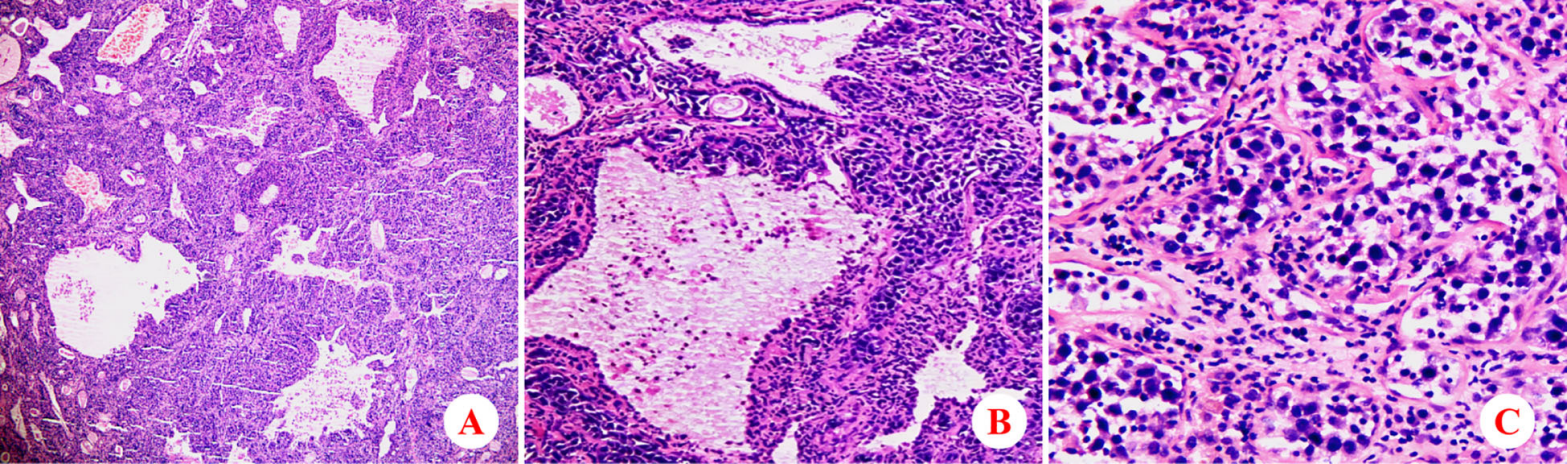
**(A)** Tumor cells accumulate in the thyroid follicular spaces, with no clear demarcation or transitional zone from normal tissue, resembling lymphocytic infiltration 40×. **(B)** Tumor cells are present within irregular capillaries 100×. **(C)** The lumen is filled with atypical large lymphoid cells; the vascular wall is intact, with no obvious extravascular invasion 200×.

**Figure 3:**
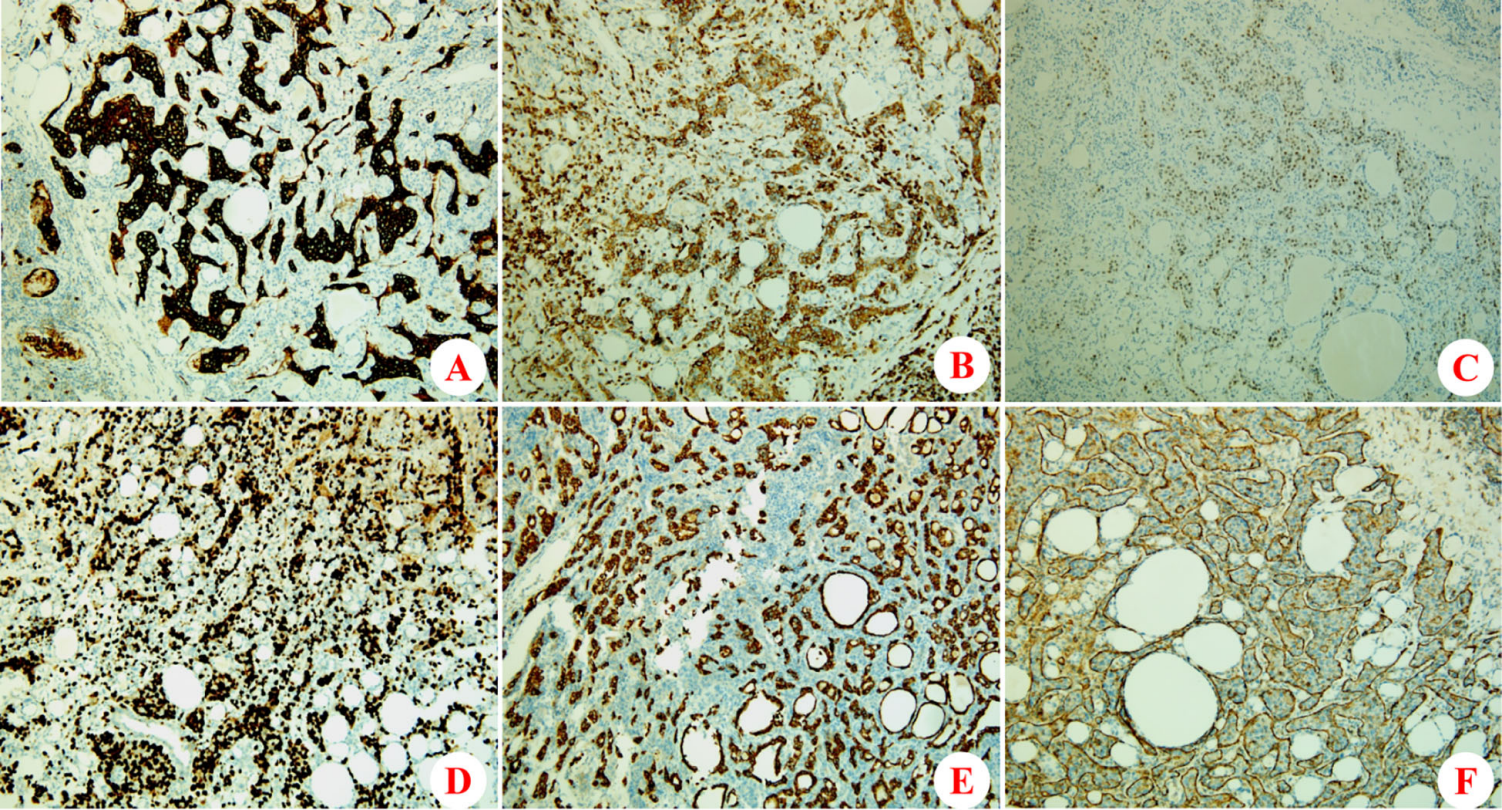
**(A)** Tumor cells show diffuse positive staining for CD20 100×. **(B)** Tumor cells show diffuse positive staining for CD5 100×. **(C)** Tumor cells show diffuse positive staining for MUM1 100×. **(D)** The proliferation index (Ki-67) is 80% 100×. **(E)** Thyroid follicular epithelial cells show diffuse positive staining for CKpan 100×. **(F)** Capillary endothelial cells are positive for CD31 100×.

Microscopically, under low magnification, scattered tumor cells were observed in the interfollicular spaces of the thyroid gland against the background of nodular goiter, mimicking benign lymphocytic infiltration (**Figure 2A**). At high magnification, increased small vessels with dilated lumina were noted in the thyroid interfollicular spaces, and the lumina were filled with atypical large lymphoid cells. The vascular walls remained intact, without obvious extravascular infiltration (**Figures 2B, C**). The neoplastic cells were large, with hyperchromatic and irregular nuclei, prominent nucleoli, and frequent mitotic figures. Immunohistochemical examination revealed that the tumor cells were positive for CD20 (**Figure 3A**), CD79a, PAX5, CD5 (**Figure 3B**), and MUM-1 (**Figure 3C**). The positive rate of c-Myc was approximately 50%, that of p53 was about 5%, and the Ki-67 proliferation index was 80% (**Figure 3D**). The tumor cells were negative for CD3, CD10, BCL6, BCL2, CD30, CD56, synaptophysin (Syn), chromogranin A (CgA), CKpan (**Figure 3E**), PAX8, and TTF-1. All neoplastic cells were located within the lumina of small vessels highlighted by CD31 (**Figure 3F**). Circulating tumor cells were negative. *In situ* hybridization demonstrated negative EBER expression.

Combined with the above clinical and pathological features, the patient was finally diagnosed with bilateral thyroid intravascular large B-cell lymphoma (IVLBCL), with a non-germinal center B-cell (non-GCB) immunophenotype. The tumor was predominantly confined within the lesions of nodular goiter, without invasion into the surrounding normal thyroid parenchyma or cervical lymph nodes. Subsequent bone marrow biopsy revealed no lymphoma infiltration. Postoperative whole-body PET-CT indicated bilateral thyroid absence was noted, and local radioactive uptake was consistent with postoperative changes. Two slightly hypodense nodules in the left hepatic lobe, a left adrenal nodule, and multiple nodules in the right inguinal region were highly suspected of lymphoma infiltration. According to the 2022 Lugano lymphoma staging system, the case was classified as stage IVA. The 63-year-old patient presented with stage IV disease involving three extranodal organs. Comprehensive assessment based on the International Prognostic Index (IPI) for diffuse large B-cell lymphoma yielded an IPI score of 3 points, corresponding to the intermediate-high risk prognostic stratification. Peripheral blood CTC testing was performed for individualized comprehensive assessment considering concurrent hepatic and adrenal involvement of the patient, to facilitate evaluation of systemic tumor burden. A negative CTC result meant no intact viable lymphoma cells were detected in peripheral blood samples. This result is in line with the pathological features of intravascular large B-cell lymphoma, as tumor cells proliferate predominantly within organ microvascular lumens and rarely shed into peripheral blood circulation.

The patient received 8 cycles of R-CHOP regimen (rituximab, cyclophosphamide, doxorubicin, vincristine, and prednisone), combined with lenalidomide to prevent central nervous system metastasis, and the disease was well controlled. Imaging examinations and laboratory parameters were regularly reviewed every two chemotherapy cycles to dynamically evaluate therapeutic response, monitor treatment-related adverse reactions, and guide individualized adjustment of the treatment strategy. The complete diagnostic profile and full clinical timeline of this case are summarized in **Table 1**.

**Table 1:** Clinical timeline of the patient with intravascular large B-cell lymphoma (IVLBL).

Date	Event	Key findings
2024-04-27	Physical Examination	Bilateral thyroid enlargement detected
2024-04-30	Thyroid Ultrasound	Bilateral thyroid lesions, TI-RADS 4 (left 4B, right 4A).
2024-05-09	Surgery	Bilateral thyroidectomy with central lymph node dissection performed.
2024-05-16	Pathological diagnosis	Intravascular large B-cell lymphoma (IVLBCL) of bilateral thyroid.
2024-05-20	PET-CT	Postoperative thyroid changes plus multi-site lesions (liver, adrenal gland, inguinal nodes), consistent with lymphoma infiltration.
2024-05-21	Treatment	Systemic therapy with 8 planned cycles of R-CHOP plus lenalidomide initiated.
2024-07-05	Cycle 2 chemotherapy	Mild anemia; persistent lesions in the liver and adrenal glands; right inguinal lymphadenopathy
2024-08-25	Cycle 4 chemotherapy	Persistent mild anemia; stable lesions in liver and adrenal glands; no obvious inguinal lymphadenopathy.
2024-10-15	Cycle 6 chemotherapy	Persistent mild anemia, LDH 515 U/L; hepatic lesions decreased significantly.
2025-01-03	8 cycles of chemotherapy	PET-CT confirmed complete remission (CR), with a Deauville score of 1.
2026-02-21	telephone follow-up	The patient died of disease relapse

TI-RADS, Thyroid Imaging Reporting and Data System; LDH, lactate dehydrogenase; CR, complete metabolic remission.

## Discussion

Lymphoma arising in the thyroid gland is rare, accounting for approximately 1%–5% of all thyroid malignancies and 2%–5% of extranodal lymphomas. The most common subtype is diffuse large B-cell lymphoma, accounting for about 60%–70%, followed by marginal zone lymphoma at approximately 30%–40% (5). Rare subtypes include follicular lymphoma, Hodgkin lymphoma, T/NK-cell lymphoma, and others. Among these, intravascular large B-cell lymphoma (IVLBCL) is extremely rare, although several instances of thyroid involvement by IVLBCL have been reported (6–13), most of these studies provide only limited descriptions with subtle clinical manifestations. Notably, when IVLBCL occurs in the setting of nodular goiter or is accompanied by other concurrent thyroid malignancies, it can be easily missed even by experienced pathologists, representing a significant diagnostic challenge. The patient in this study presented with concurrent IVLBCL and nodular goiter. Neoplastic cells showed localized growth within thyroid interfollicular stroma and closely mimicked interstitial inflammatory changes, rendering the lesion easily overlooked at low magnification.

IVLBCL has an extremely diverse and nonspecific clinical presentation. Based on involved sites and clinical features, IVLBCL is classified into the classical type and the Asian variant (14–16). The classical type is mainly manifested by central nervous system damage or cutaneous involvement, whereas the Asian variant is characterized by hemophagocytic syndrome, frequently involving the liver, spleen and bone marrow sinusoids, and clinically presenting with fever, anemia, hepatosplenomegaly and other symptoms. In addition, bone marrow biopsy is conducive to the accurate differentiation of the two clinical phenotypes (17). IVLBCL generally does not form solid tumors. It presents non-specific imaging features and lacks sensitive early diagnostic methods, which easily results in delayed diagnosis and treatment in some patients; a few cases are only discovered at autopsy. Ponzoni et al. (4) reported that laboratory abnormalities, including elevated serum lactate dehydrogenase, anemia, thrombocytopenia and hypoalbuminemia, are suggestive of this disease. At the initial visit, the patient only presented with isolated night sweats without fever, and had no manifestations related to hemophagocytic syndrome or bone marrow involvement. Postoperative PET-CT revealed multisystem involvement. In addition to the thyroid gland, abnormal lesions were observed in the liver, adrenal glands and inguinal region, while no evidence of central nervous system involvement was found. Collectively, this represents a clinically rare and atypical case. Combined with clinical characteristics, imaging findings and pathological morphology, this case was preferentially classified as the Asian variant of IVLBCL.

IVLBCL is a distinctive non-epithelial B-cell neoplasm characterized by tumor cells predominantly proliferating within vascular lumens and constitutive loss of EpCAM (CD326) expression. The reported positive rate of circulating tumor cells (CTCs) in IVLBCL is merely 10%–15%, predominantly seen in patients with bone marrow involvement and high tumor burden (18, 19). Given the multiple organ involvement identified on PET-CT in this patient, CTC testing was carried out and returned a negative result. Although this finding suggests no obvious hematogenous dissemination, it should be noted that the absence of EpCAM in IVLBCL cells compromises the performance of mainstream EpCAM-based CTC assays including the CellSearch system, which may give rise to false-negative readings (18). Clinically, CTCs serve as valuable liquid biopsy biomarkers to reflect hematogenous spread, assist disease staging, guide treatment strategies, and assess therapeutic response and long-term prognosis.

Of particular interest, the present case showed IVLBCL confined exclusively to the area of nodular goiter, with no evidence of tumor invasion in the surrounding normal thyroid tissue. This distinctive growth pattern is extremely rare, and its underlying mechanism remains unclear. Studies have shown that the characteristic intravascular distribution of IVLBCL is closely related to the expression of CXCR3 on the surface of tumor cells, which exhibits high affinity for CXCL9 secreted by vascular endothelial cells (20). Other studies have reported that tumor cells lack ICAM-1, β1-integrin, LFA-1 (lymphocyte function-associated antigen-1), or other molecules involved in transvascular migration and lymphocyte homing (21, 22), may also contribute to the preferential localization of tumor cells within vascular lumens. Katalinic et al. (7) suggested that the rich vascular supply of the thyroid gland and adenomatous nodule microenvironment may induce overexpression of these chemokines on endothelial cells locally, leading to early preclinical thyroid gland involvement and incidental diagnosis in asymptomatic patients. Consistent with the above findings, the present patient only presented with mild nocturnal sweating and had no specific clinical manifestations. The patient initially sought medical treatment for multinodular goiter and was eventually diagnosed with occult IVLBCL by chance. This case further suggests that the unique microenvironment of thyroid nodules serves as a potential predisposing condition for intravascular lymphoma. Tumors tend to primarily involve nodular lesions, which mechanistically explains why the lesion in this case was confined solely to thyroid nodules. Meanwhile, clinicians and pathologists should maintain high vigilance and strengthen the differential diagnosis of such rare and easily missed diseases.

Due to the characteristic intravascular growth pattern of tumor cells in IVLBCL, no distinct mass formation is observed either grossly or microscopically, which poses a diagnostic challenge. The tumor cells are positive for B-lymphocyte–associated antigens including CD20, CD79a, and PAX5, which allows differentiation from intravascular T-cell and intravascular NK-cell lymphomas. It should also be distinguished from other B-cell immunophenotypic intravascular lymphoproliferative disorders, such as intravascular large B-cell lymphoma with plasmacytic differentiation and benign atypical intravascular lymphoid hyperplasia, as well as benign lesions including intravascular CD30-positive T-cell proliferation and intravascular lymphohistiocytosis. Additionally, differential diagnosis includes intravascular metastatic carcinoma and angiosarcoma. In the present study, the diagnosis of IVLBCL was confirmed based on combined histomorphological features and immunohistochemical staining results.

IVLBCL is highly aggressive with an overall poor prognosis, and the median survival time is approximately 1–2 years. One study indicated that age < 70 years, initial diagnosis at non-central nervous system sites, LDH < 700 U/L, and treatment with rituximab were favorable prognostic factors (2). In contrast, patients with hemophagocytic syndrome or CNS involvement have a worse prognosis. Other studies have shown that CD5-positive IVLBCL is more aggressive than CD5-negative disease, is more frequently accompanied by bone marrow and peripheral blood abnormalities, and is associated with a poorer prognosis (23, 24). In the present patient, diffuse CD5 positivity represents an adverse prognostic factor. In addition, several other potential prognostic factors may be involved, such as general physical status and extravascular involvement.

R-CHOP (rituximab, cyclophosphamide, doxorubicin, vincristine and prednisone) remains the standard first-line regimen for IVLBCL, wherein rituximab exerts antitumor activity by targeting CD20 on B cells and potentiating chemotherapeutic cytotoxicity. We administered lenalidomide combined with R-CHOP (R2-CHOP) to our patient with thyroid IVLBCL, a therapeutic choice backed by subtype-specific immunomodulatory benefits validated in multiple prospective clinical trials (25). Randomized phase II and III studies confirmed that IVLBCL predominantly belongs to ABC-subtype DLBCL, and the addition of lenalidomide yields prominent clinical benefits. Mechanistically, lenalidomide alleviates microvascular immune suppression, downregulates tumoral PD-L1 expression, and enhances rituximab-mediated ADCC, which targets the characteristic intravascular growth pattern of this rare lymphoma (26). This risk is particularly prominent in patients with thyroid IVLBCL obscured by benign thyroid lesions including multinodular goiter. As summarized in the 2024 Chinese expert consensus, this regimen exhibits satisfactory safety in patients complicated with chronic thyroid diseases without exacerbating thyroid dysfunction, which rationalizes the treatment selection for our patient (27). Nevertheless, several limitations exist: unified predictive biomarkers for screening patients who derive maximal benefit from lenalidomide have not been established, and long-term data regarding thyroid-origin IVLBCL are still limited, warranting additional real-world investigations.

Notably, favorable disease control achieved by R2-CHOP could not be maintained in our case due to inadequate follow-up. Long-term follow-up demonstrated that the patient could not complete regular surveillance and maintenance therapy owing to economic hardship. The patient eventually died of disease recurrence and progression, accompanied by systemic involvement manifestations including fever, headache and dyspnea before death. This clinical course further illustrates the highly aggressive biological behavior and high recurrence propensity of IVLBCL.

## Conclusion

In summary, IVLBCL arising within multinodular goiter is an extremely rare and easily missed disease. Its non-specific clinical manifestations and imaging features pose significant diagnostic challenges to pathologists and clinicians. Immunohistochemistry is crucial for an accurate diagnosis. Early recognition and timely chemotherapy with the R-CHOP regimen can effectively improve patient prognosis. Enhancing awareness of this rare disease is the key to avoiding missed diagnosis.

## Data Availability

The original contributions presented in the study are included in the article/supplementary material. Further inquiries can be directed to the corresponding author.
